# Prognostic Significance of Sarcopenia With Inflammation in Patients With Head and Neck Cancer Who Underwent Definitive Chemoradiotherapy

**DOI:** 10.3389/fonc.2018.00457

**Published:** 2018-10-22

**Authors:** Yeona Cho, Jun Won Kim, Ki Chang Keum, Chang Geol Lee, Hei Cheul Jeung, Ik Jae Lee

**Affiliations:** ^1^Department of Radiation Oncology, Gangnam Severance Hospital, Yonsei University College of Medicine, Seoul, South Korea; ^2^Department of Radiation Oncology, Yonsei Cancer Center, Yonsei University College of Medicine, Seoul, South Korea; ^3^Division of Medical Oncology, Gangnam Severance Hospital, Yonsei University College of Medicine, Seoul, South Korea

**Keywords:** sarcopenia, inflammation, head and neck cancer, chemoradiotherapy, survival

## Abstract

**Purpose:** With growing evidence that inflammation and low muscularity play a role in the survival of cancer patients, we evaluated the prognostic implications of sarcopenia with systemic inflammation in patients who underwent definitive chemoradiotherapy (CCRT) for locally advanced head and neck cancer.

**Materials and Methods:** We analyzed 221 patients with head and neck cancer who received definitive CCRT between 2006 and 2015. The skeletal muscle area was measured using a single computed tomography image slice at the level of the third lumbar vertebra (L3). Sarcopenia was defined as an L3 muscle index of <49 cm^2^/m^2^ for men and <31 cm^2^/m^2^ for women.

**Results:** Patients with sarcopenia (*n* = 106) exhibited higher neutrophil/lymphocyte ratios (NLRs) than those without (*n* = 115); the former also had an inferior 3-year overall survival (OS) rate (62%) than the latter (76%, *p* = 0.037). Among patients with sarcopenia, those who also had high NLRs (*n* = 51) showed significantly poorer OS and progression-free survival (PFS). In the multivariate analysis, sarcopenia plus a high NLR remained the most significant predictor of poor OS and PFS. Patients with sarcopenia required more frequent interruption of RT; patients whose RT was interrupted for ≥5 days showed inferior disease control and OS.

**Conclusions:** Sarcopenia accompanied by systemic inflammation at initial diagnosis is associated with significantly inferior OS and PFS. Additionally, patients with sarcopenia required RT interruption more frequently. Intensive nutritional support and additional treatment methods are required for these patients while undergoing RT.

## Introduction

Patients with head and neck cancer may experience progressive weight loss following their diagnosis; significant weight loss is associated with poorer overall survival (OS) (1, 2). The prognoses of these patients can be influenced in part by changes in body composition and loss of body weight (3). Cancer cachexia is related to the activation of systemic inflammatory responses as well as imbalances in energy intake and expenditure over the disease course and during treatment. During this process, sarcopenia is the most commonly occurring condition (4, 5).

Sarcopenia, the loss of skeletal muscle mass, function, and strength, has emerged as an important prognostic factor in various types of cancer, including head and neck cancer (6). It is associated with an older age, poor performance status, malnutrition, and treatment-related toxicities. However, emerging evidence suggests that sarcopenia is a prevalent condition in cancer patients regardless of disease stage and nutritional status and is associated with higher mortality rates and/or poor disease control (7).

Meanwhile, numerous studies have suggested that systemic inflammation is indicative of cancer aggressiveness and is associated with poor prognoses. The neutrophil/lymphocyte ratio (NLR) is widely used as a marker of systemic inflammation (8). Patients with different types of cancer who exhibit elevated NLRs show very poor disease control as well as inferior OS. Previous studies have emphasized the role of systemic inflammation as a driver of muscle degradation in cancer patients (9, 10). Patients with colorectal cancer and high NLRs have a significantly lower skeletal muscle index, and those with both high NLRs and sarcopenia show inferior OS rates (11). Male patients with small cell lung cancer and sarcopenia accompanied by high NLRs were shown to have poorer prognoses and not tolerate standard treatments (12).

Previous studies of sarcopenia in cancer patients focused on the effect of surgery and/or chemotherapy; there are limited data on the effect of sarcopenia combined with systemic inflammation in patients with head and neck cancer who undergo definitive chemoradiotherapy (CCRT), including CCRT-related toxicities and tolerance. Therefore, we hypothesized that sarcopenia, especially when accompanied by systemic inflammation, plays a significant role in determining survival, disease control, and treatment tolerance in patients undergoing CCRT for head and neck cancer.

## Methods

### Patients and treatment profiles

In a single-institutional retrospective study, we analyzed 272 patients with locally advanced (American Joint Committee on Cancer [AJCC] stage III–IVB) head and neck cancer, including cancer of the oral cavity, nasopharynx, oropharynx hypopharynx, and larynx, who received definitive radiotherapy (RT) between January 2006 and December 2015. Patients who had undergone whole-body positron emission tomography (PET)-computed tomography (CT) or abdominal CT at diagnosis were eligible for the study. Those who had no CT data of sufficient quality to perform accurate measurements of the tissue area at the 3rd lumbar level (L3) (*n* = 43) or no height data (*n* = 8) were excluded; hence, our final cohort comprised 221 patients. The body mass index (BMI) grouping based on the World Health Organization criteria is as follows: BMI < 18.5 kg/m^2^ (underweight), BMI 18.5–25 kg/m^2^ (normal weight), BMI 25–30 kg/m^2^ (overweight), and BMI ≥ 30 kg/m^2^ (obese). This study was approved by the institutional review board of the Gangnam Severance Hospital (protocol number 3-2017-0269).

Patients were treated with definitive RT alone, concurrent chemoradiotherapy (CCRT), or induction chemotherapy followed by CCRT depending on the primary tumor site, tumor stage, risk factors, and/or the physicians' decisions. External beam RT was performed with 3D-conformal RT (54–70.2 Gy) or intensity-modulated RT (IMRT) (55.2–74.2 Gy).

### Measurement of body composition and definition of sarcopenia

**Figure 1:**
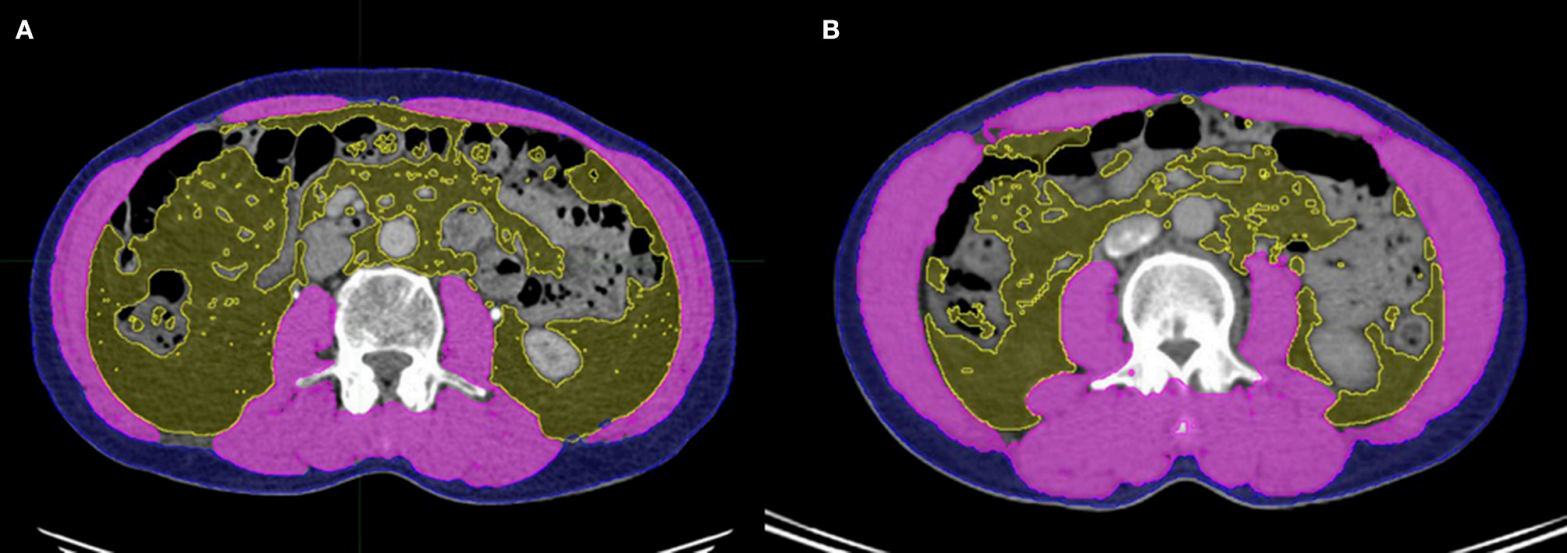
Slices of computed tomography (CT) images of patients with **(A)** and without **(B)** sarcopenia who had similar body mass indices. Skeletal muscle, visceral adipose tissue, and subcutaneous adipose tissue were measured using the MIM Vista software (MIM corp., version 6.1, OH, USA).

Because dual-energy X-ray absorptiometry (DXA) is infrequently used in routine clinical practice, we used a previously validated CT-based body composition method with scans acquired at the initial diagnosis (i.e., whole-body PET-CT or abdominal CT). For each CT scan, a single axial slice at the level of L3 was selected. The MIM Vista software (MIM corp., Version 6.1, OH, USA) was used to demarcate skeletal muscle, visceral fat tissue, and subcutaneous fat tissue according to predefined validated boundaries based on Hounsfield units (HUs). The following thresholds were applied: −29 to +150 HU for skeletal muscle, −150 to −50 HU for visceral fat tissue, and −190 to −30 HU for subcutaneous fat tissue. Figure 1 represents the slices of computed tomography (CT) images of patients with (A) and without (B) sarcopenia who had similar body mass indices. The radiation oncologist who performed these measurements was blinded to the treatment outcomes of all patients to minimize bias.

The cross-sectional area of muscle and fat tissue (in square centimeters) was normalized to the square of height in meters and reported as the skeletal muscle index (cm^2^/m^2^). According to international consensus, sarcopenia is defined as an L3 muscle index of below 55 cm^2^/m^2^ in men and below 39 cm^2^/m^2^ in women (13). In this study of Korean patients, however, we defined sarcopenia as an L3 muscle index of below 49 cm^2^/m^2^ for men and below 31 cm^2^/m^2^ for women, based on a previous epidemiologic study using DXA and the regression equation,

$$\begin{array}{l} L3\;muscle\;index\;of\;CT\;=\\ \frac{height-adjusted\;appendicular\;skeletal\;muscle\;mass\;in\;DXA\;\left(kg/m2\right)-1.17}{0.11} \end{array}$$

to convert the CT value (12, 14).

### Markers of systemic inflammation

The patients' blood counts were obtained prior to performing diagnostic procedures or administering treatments. Our primary determinant of systemic inflammation was the NLR obtained at the initial diagnosis. Receiver Operating Characteristic (ROC) curves were constructed for the NLR using OS as the primary endpoint. A high NLR was defined as ≥2.7 with an area under the curve of 0.66, with a sensitivity of 57.9% and specificity of 72.8% (Supplementary Figure 1), according to a previous meta-analysis of the NLR in head and neck cancer that revealed cutoff values of the NLR for dichotomization ranging from 1.92 to 5 (median, 2.69) (8). Data on serum albumin levels, which are of particular interest as a marker of both nutritional status as well as systemic inflammation, were available for 218 participants.

### Statistical analysis

Categorical data were analyzed using the Fisher's exact test or χ^2^ analyses, while continuous data between groups were compared using the Mann-Whitney-*U*-test. OS was defined as the interval between diagnosis and death from any cause or last follow-up. Progression-free survival (PFS) was defined as the interval between diagnosis to the detection of first progression, death from any cause, or last follow-up. The Kaplan-Meier method and log-rank test were used to estimate and compare OS and PFS rates. We obtained hazard ratios (HRs) using the cumulative survivor function. Univariate and multivariate analyses for OS and PFS were conducted with the Cox proportional hazards model. We performed multivariate Cox hazard regression analysis on the variables that showed *p* < 0.05 in the univariate analysis. HRs and their corresponding 95% confidence intervals (CIs) are reported. *P* < 0.05 was considered statistically significant. All analyses were performed with IBM SPSS version 20.0 (SPSS, Chicago, IL, USA).

## Results

### Patient and treatment characteristics

Between 2006 and 2015, a total of 221 patients with head and neck cancer (74, 58, 50, 32, and 7 patients with nasopharyngeal, oropharyngeal, hypopharyngeal, laryngeal, and oral cavity cancer, respectively) received definitive RT (Table 1). The median age at diagnosis was 59 years (range 18–94 years); 180 patients (81.4%) were men and 41 (18.6%) women. Most patients received CCRT (92.3%; of these, 71.9% underwent only CCRT whereas 20.4% underwent induction chemotherapy followed by CCRT), while only 7.7% received RT alone. The median BMI was 22.6 kg/m^2^, and the mean NLR was 2.55 (range 0.78–24.47).

**Table 1:** Patient characteristics.

		**Total**		**Non-sarcopenia**		**Sarcopenia**		***p*-value**
**Characteristics**		***N* = 221**	**%**	***N* = 115**	**%**	***N* = 106**	**%**	
Age	Median	59		56		64		0.01
	(Range)	18–94		18–86		18–94		
Sex	Male	180	81.4	76	66.1	104	98.1	<0.001
	Female	41	18.6	39	33.9	2	1.9	
^a^ECOG PS	0.1	177	80.1	106	92.2	71	67.0	<0.001
	≥2	44	19.9	9	7.8	35	33.0	
Primary site	Nasopharynx	74	33.5	41	35.7	33	31.1	0.443
	Oropharynx	58	26.2	33	28.7	25	23.6	
	Hypopharynx	50	22.6	22	19.1	28	26.4	
	Larynx	32	14.5	17	14.8	15	14.2	
	Oral cavity	7	3.2	2	1.7	5	4.7	
T stage	T1	27	12.2	16	13.9	11	10.4	0.034
	T2	48	21.7	29	25.2	19	17.9	
	T3	63	28.5	23	20.0	40	37.7	
	T4	83	37.6	47	40.9	36	34.0	
N stage	N0	35	15.8	17	14.8	18	17.0	0.958
	N1	30	13.6	15	13.0	15	14.2	
	N2	145	65.6	77	67.0	68	64.2	
	N3	11	5.0	6	5.2	5	4.7	
^b^AJCC stage	III	70	31.7	33	28.7	37	34.9	0.321
	IVA/B	151	68.3	82	71.3	69	65.1	
^c^BMI	Underweight	23	10.4	5	4.3	18	17.0	<0.001
	Normal weight	153	69.2	77	67.0	76	71.7	
	Overweight	42	19.0	30	26.1	12	11.3	
	Obese	3	1.4	3	2.6	0	0.0	
^d^SMI	Median	46.4		51.1		43.2		<0.001
(cm^2^/m^2^)	(Range)	4.9–88.0		31.5–88.0		4.93–48.9		
^e^VFI	Median	79.5		86.1		70.8		0.089
(cm^2^/m^2^)	(Range)	0.5–305.8		3.3–305.8		0.5–301.3		
^f^SFI	Median	91.7		106.8		71.4		<0.001
(cm^2^/m^2^)	(Range)	0.4–289.0		8.3–271.4		0.4–289.0		
Hemoglobin	Mean	13.7		13.8		13.4		0.045
(g/dL)	(Range)	7.5–17.1		8.6–17.1		7.5–16.5		
^g^WBC	Mean	7		7.1		7.9		0.023
(x10^3^ cells/μL)	(Range)	3.0–22.3		3.3–13.4		3.0–22.3		
^h^ANC	Mean	4.5		4.6		5.3		0.023
(x10^3^ cells/ μL)	(Range)	1.4–20.7		1.6–11.3		1.4–20.7		
^i^NLR	Mean	2.55		2.86		3.5		0.053
	(Range)	0.78–24.47		0.84–11.06		0.78–24.47		
Platelets	Mean	254		265		267		0.88
(x10^3^ cells/μL)	(Range)	40–563		82–526		40–563		
Albumin	Mean	4.3		4.3		4.2		0.108
(g/dL)	(Range)	3.0–5.1		3.0–5.1		3.1–4.8		

a *ECOG PS, Eastern Cooperative Oncology Group performance status*.

b *AJCC, American Joint Committee on Cancer*.

c *BMI, body mass index*.

d *SMI, skeletal muscle index*.

e *VFI, visceral fat index*.

f *SFI, subcutaneous fat index*.

g *WBC, white blood cell count*.

h *ANC, absolute neutrophil count*.

i *NLR, neutrophil/lymphocyte ratio*.

Patients were divided into 2 groups (those with vs. those without sarcopenia), based on the L3 muscle index cut-off described in the Materials and Methods section. The characteristics of the patients in the non-sarcopenia group (*n* = 115, 52%) and sarcopenia group (*n* = 106, 48%) are shown in Table 1. The median skeletal muscle indices were 46.4 (range 4.9–88.0), 51.1 (range 31.5–88.0), and 43.2 (range 4.93–48.9) in the total cohort, non-sarcopenia, and sarcopenia group, respectively. Patients in the sarcopenia group were older, predominantly male (98.1%), and exhibited a poorer performance status and more advanced T-stage disease than those in the non-sarcopenia group. The proportion of patients who were underweight was higher in the sarcopenia than in the non-sarcopenia group (17.0 vs. 4.3%, *p* < 0.001); patients in the sarcopenia group also had lower visceral and subcutaneous fat indices. When considering inflammatory markers, elevated white blood cell (WBC) counts and absolute neutrophil counts (ANCs) were observed in patients with sarcopenia; moreover, the NLR tended to be higher in this group (mean: 3.5 vs. 2.86, *p* = 0.053). Serum albumin levels were similar in both groups.

### Treatment profile and toxicity analysis

Most patients received CCRT (*n* = 204, 92.3%), and 82.4% underwent IMRT. More than half of the patients received a radiation dose of 70 Gy or higher. The treatment schemes, RT modalities, RT doses, and fraction sizes were not significantly different between the 2 groups (Table 2), nor were the total RT times (49 vs. 51 days, *p* = 0.686) or treatment completion rates. However, RT interruption of ≥5 days was more frequent in the sarcopenia than in the non-sarcopenia group (18.9 vs. 7.8%, *p* = 0.015).

**Table 2:** Treatment profile and toxicity analysis.

		**Total**		**Non-sarcopenia**		**Sarcopenia**		***p*-value**
**Characteristics**		***N* = 221**	**%**	***N* = 115**	**%**	***N* = 106**	**%**	
Treatment scheme	^a^RT alone	17	7.7	6	5.2	11	10.4	0.286
	^b^CCRT	159	71.9	83	72.2	76	71.7	
	Induction + CCRT	45	20.4	26	22.6	19	17.9	
RT modality	^c^3D-CRT	39	17.6	18	15.7	21	19.8	0.418
	^d^IMRT	182	82.4	97	84.3	85	80.2	
RT dose	< 70 Gy	96	43.4	47	40.9	49	46.2	0.422
(^e^EQD2, α/β = 3)	≥70 Gy	125	56.6	68	59.1	57	53.8	
Fraction size	< 210	59	26.7	31	27.0	28	26.4	0.928
(cGy)	≥210	162	73.3	84	73.0	78	73.6	
RT time	Median	50		49		51		0.686
(days)	(Range)	(30–94)		(30–71)		(31–94)		
Interruption of RT	No	194	87.8	106	92.2	86	81.1	0.015
(≥5 days)	Yes	27	12.2	9	7.8	20	18.9	
Completion of RT	No	10	4.5	6	5.2	4	3.8	0.75
	Yes	211	95.5	109	94.8	102	96.2	
Acute toxicity	All	71	32.1	40	34.8	31	29.2	0.378
(^f^RTOG Grade≥3)	^g^A/N/V	10	4.5	0	0.0	1	0.9	0.081
	Dysphagia	3	1.4	1	0.9	2	1.9	0.863
	Skin reaction	38	17.2	6	5.2	7	6.6	0.778
	Oral mucositis	48	21.7	31	27.0	16	15.1	0.031
	Pharyngitis	19	8.6	8	7.0	11	10.4	0.365

a *RT, radiotherapy*.

b *CCRT, concurrent chemoradiotherapy*.

c *3D-CRT, 3-dimensional conformal radiotherapy*.

d *IMRT, intensity-modulated radiotherapy*.

e *EQD2: equivalent dose in 2 Gy*.

f *RTOG, Radiation Therapy Oncology Group*.

g *A/N/V, anorexia/nausea/vomiting*.

Acute toxicity was evaluated according to the Radiation Therapy Oncology Group toxicity grading criteria. Seventy-one patients experienced grade (G) 3 acute toxicity, while none had G4 or G5 acute toxicities. No patient exhibited ≥G3 fatigue, and only 1 had G3 anorexia/nausea/vomiting. Dysphagia, dry mouth, skin reaction, and pharyngitis were observed at similar rates in both groups. However, ≥G3 oral mucositis was more frequent in the non-sarcopenia than in the sarcopenia group.

### Analysis of survival and prognostic factors

**Figure 2:**
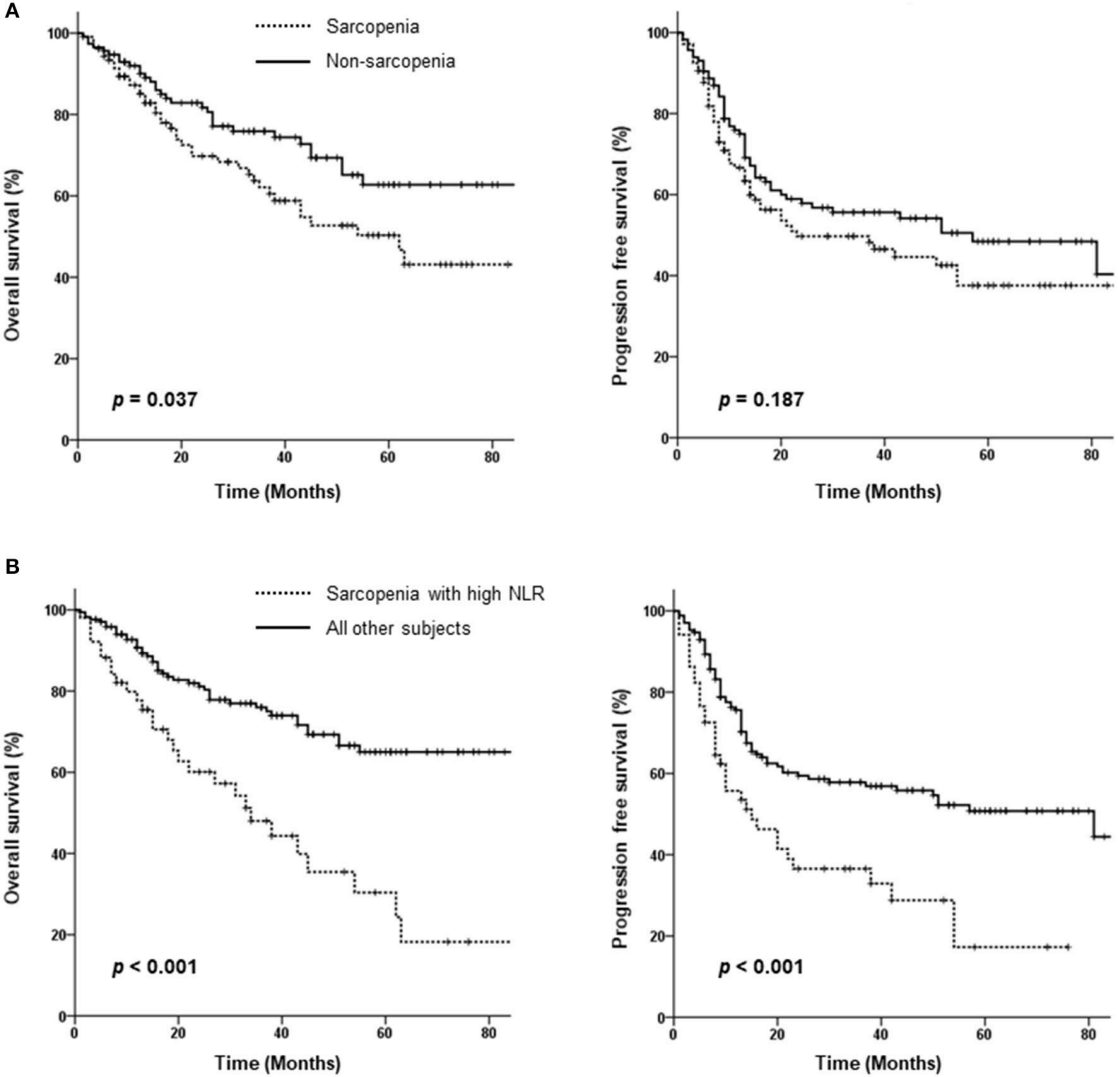
Kaplan-Meier curves comparing overall and progression-free survival between the sarcopenia and non-sarcopenia groups **(A)** as well as between patients with sarcopenia plus a high neutrophil/lymphocyte ratio (NLR) and those without sarcopenia or a low NLR **(B)**.

Seventy-one patients died over a median follow-up duration of 30 months (range 1–110 months). Fifty-nine patients experienced loco-regional failure, and 28 developed distant metastasis. The 3-year OS and PFS rates among all 221 patients were 69.6 and 52.9%, respectively. As shown in Figure 2A, patients in the sarcopenia group showed poorer OS than those in the non-sarcopenia group (3-year OS: 62 vs. 76%, *p* = 0.037); however, PFS rates were not significantly different between the 2 groups (3-year PFS: 46.6 vs. 55.6%, *p* = 0.187).

As sarcopenia is associated with cancer-related inflammation, we assessed the effect of sarcopenia with inflammation on survival using the NLR as a marker of systemic inflammation. An of NLR ≥2.7 was defined as high; survival was compared between patients with sarcopenia plus high NLR vs. others (those with no sarcopenia or a low NLR). Patients with sarcopenia and high NLRs exhibited significantly lower PFS (3-year PFS: 36.5 vs. 57.8%, *p* < 0.001) and poorer OS (3-year OS: 48 vs. 76%, *p* < 0.001) (Figure 2B). Both locoregional failure and distant metastasis were more frequently observed in the sarcopenia plus high NLR group (64.7 vs. 39.4%, *p* < 0.001 and 58.8 vs. 30%, *p* < 0.001, respectively).

In the univariate analysis, sarcopenia, the NLR, and sarcopenia plus a high NLR were significantly associated with OS and PFS. As these 3 variables are highly correlated with another, we first conducted a multivariate analysis including the two variables sarcopenia and NLR. A high NLR but not sarcopenia was significantly associated with poor OS (HR 2.240, 95% CI 1.344–3.732, *p* = 0.002) and PFS (HR 1.715, 95% CI 1.100–2.673, *p* < 0.001). Second, we performed a multivariate analysis using the variable “sarcopenia and NLR status,”; this analysis showed that sarcopenia plus a high NLR was a significant prognostic factor for OS and PFS (OS: HR 2.785, 95% CI 1.674–4.634, *p* < 0.001; PFS: HR 2.145, 95% CI 1.429–3.220, *p* < 0.001). Taken together, sarcopenia plus high NLR was the most significant predictor of poor OS and PFS (Table 3). Older age was a significant prognostic factor for OS, and a primary oral cavity cancer and advanced stage were predictors of both poor PFS and OS.

**Table 3:** Univariate and multivariate analysis for overall survival (OS) and progression-free survival (PFS).

		**OS**			**OS**			**PFS**			**PFS**		
**Variable**		**Univariate analysis**			**Multivariate analysis**			**Univariate analysis**			**Multivariate analysis**		
		**HR**	**95%CI**	***p*-value**	**HR**	**95%CI**	***p*-value**	**HR**	**95%CI**	***p* value**	**HR**	**95%CI**	***p*-value**
Age	< 60 years	1			1			1			1		
	≥60 years	2.504	1.551–4.043	<0.001	1.730	1.008–2.968	0.047	2.056	1.395–3.032	<0.001	1.392	0.898–2.160	0.898
Sex	Male	1						1					
	Female	0.962	0.527–1.756	0.900				0.919	0.056–1.510	0.738			
Primary site	Nasopharynx	1			1			1			1		
	Oropharynx	2.200	1.150–4.207	0.017	1.337	0.638–2.802	0.442	1.845	1.093–3.116	0.022	1.222	0.676–2.207	0.507
	Hypopharynx	3.371	1.752–6.486	<0.001	1.630	0.763–3.480	0.207	2.810	1.660–4.758	<0.001	1.656	0.897–3.057	0.107
	Larynx	1.985	0.882–4.469	0.098	1.546	0.648–3.689	0.326	2.006	1.064–3.783	0.032	1.676	0.847–3.318	0.138
	Oral cavity	7.914	2.900–21.6	<0.001	5.784	2.030–16.480	0.001	5.101	1.949–3.347	0.001	4.177	1.517–1.498	0.006
AJCC stage	III	1			1			1			1		
	IVA/B	2.059	1.191–3.558	0.010	1.913	1.044–3.503	0.036	1.935	1.241–3.018	0.004	1.762	1.074–2.891	0.025
Treatment	RT^a^ alone	1						1					
	^b^CCRT	0.610	0.275–1.353	0.224				0.685	0.354–1.326	0.262			
	Induction+CCRT	0.760	0.317–1.822	0.538				0.554	0.260–1.184	0.127			
RT modality	^c^3D-CRT	1						1					
	^d^IMRT	0.686	0.405–1.163	0.162				1.060	0.650–1.729	0.815			
Interruption of RT	No	1			1			1			1		
	Yes	2.084	1.161–3.742	0.014	2.122	1.142–3.941	0.017	2.084	1.161–3.742	0.014	1.773	1.040–3.023	0.035
Sarcopenia and ^e^NLR status	Non-sarcopenia or low NLR	1			1			1			1		
	Sarcopenia plus high NLR	2.187	1.746–4.547	<0.001	2.785	1.674–4.634	<0.001	2.213	1.501–3.263	<0.001	2.145	1.429–3.220	<0.001
^f^BMI	<18.5	1						1					
	≥18.5	1.293	0.560–2.986	0.547				1.12	0.599-2.094	0.723			
Anemia	No	1			1			1			1		
	Yes	1.687	1.031–2.761	0.037	0.909	0.523–1.579	0.735	1.604	1.067–2.411	0.023	1.011	0.636–1.606	0.964

a *RT, radiotherapy*.

b *CCRT, concurrent chemoradiotherapy*.

c *3D-CRT, 3-dimensional conformal radiotherapy*.

d *IMRT, intensity-modulated radiotherapy*.

e *NLR, neutrophil/lymphocyte ratio*.

f *BMI, body mass index*.

**Figure 3:**
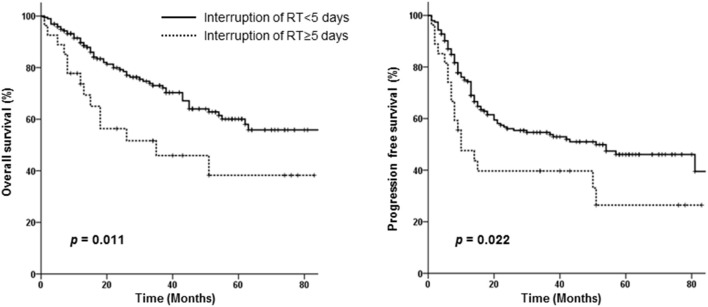
Overall and progression-free survival curves of patients requiring interruption of radiotherapy for <5 days vs. those requiring interruption for ≥5 days.

Neither the treatment scheme nor RT modality affected OS or PFS. However, an RT interruption of ≥5 days adversely affected both OS (HR 2.122, 95% CI 1.142–3.941, *p* = 0.017) and PFS (HR 1.773, 95% CI 1.040–3.023, *p* = 0.035) independently in the multivariate analysis (Table 3). Survival according to RT interruption is presented in Figure 3.

## Discussion

In this study, patients with sarcopenia had poorer OS than those without sarcopenia; furthermore, sarcopenia was closely associated with PFS and OS when accompanied by systemic inflammation. The presence of sarcopenia plus a high NLR was the most significant independent predictor of PFS and OS.

Sarcopenia alone did not decrease survival among our patients. Although it was more frequently observed among elderly patients, the development of sarcopenia may be associated with conditions that are not exclusive to older individuals. Cancer patients are generally subjected to several cancer-specific and non-cancer-specific degenerative conditions that result in decreased muscle mass and dysfunction; these include malnutrition, physical inactivity, comorbidities, and other factors directly related to disease pathophysiology and therapy-related toxicity (15). Certain types of tumors induce systemic inflammation; such cancer-related inflammation is closely associated with the progression of sarcopenia (11, 12). Patients with sarcopenia in this study exhibited increased levels of inflammatory markers such as the WBC, ANC, and NLR; this supports the notion that sarcopenia may reflect the increased metabolic activity of more aggressive tumors, leading to systemic inflammation and muscle wasting (16). Thus, we suggest that sarcopenia accompanied by systemic inflammation represents a more significant prognostic indicator.

Notably, patients with sarcopenia who also had a high NLR showed the worst prognoses; they had poor OS rates and experienced more rapid disease progression than those without sarcopenia or a high NLR. We evaluated the prognostic significance of pre-treatment sarcopenia and found it to be less related to cancer treatment or chronic malnutrition due to disease progression. These findings suggest that sarcopenia combined with a high NLR reflects a very aggressive subgroup of head and neck cancer when compared to malnutrition during the course of the disease.

Some inflammatory mechanisms implicated in cancer-related cachexia may be active in the earlier stages of disease and can lead to muscle depletion long before the appearance of extreme cancer cachexia. The direct impact of tumor-derived factors on muscle tissue, which has been explored in preclinical models that investigated the roles of tumor-induced systemic inflammation and altered metabolism (17, 18), supports this theory. Certain types of cancer can promote the up-regulation of pro-inflammatory cytokines including interleukin-6 (IL-6), tumor necrosis factor-α (TNFα), and interferon-γ, many of which are anorexigenic and/or proteolytic. Tumor factors such as the proteolysis-inducing factor are also involved in muscle atrophy in cancer patients. Both cytokines and tumor factors increase protein degradation through the ubiquitin-proteasome pathway and depress protein synthesis via the phosphorylation of eukaryotic inhibition factor 2α (19–21). Taken together, cancer-related inflammation and sarcopenia accompanying this process may reflect the aggressive nature of the tumor.

Although this study did not identify sarcopenia alone as an independent prognostic factor, previous studies suggested that sarcopenia may affect the survival, prognosis, and treatment response in cancer patients independently. Sarcopenia was shown to be associated with surgical mortality and OS in patients with esophageal, breast, colon, lung, and gastrointestinal cancers (22–25). Moreover, patients with sarcopenia were found to experience a shorter time to progression (26). The same was shown to be true for patients with head and neck cancer; Grossberg et al. revealed that skeletal muscle depletion at presentation or after treatment shortened OS and cancer-specific survival in patients with head and neck squamous cell carcinoma and sarcopenia (6). Further studies are required to identify patient subgroups in whom sarcopenia may act as an independent prognostic factor.

G3 toxicities were observed with similar frequency in both groups, although patients without sarcopenia experienced G3 oral mucositis more frequently than those with sarcopenia. Other studies found that cancer-induced cachexia and its associated pro-inflammatory cytokines may promote increased radiation-related toxicities (27, 28). However, patients with sarcopenia in our study were older and had a poorer performance status, which might have caused physicians to opt for less aggressive treatment. Nevertheless, these patients required more frequent RT interruptions than those in the non-sarcopenia group, indicating that patients with sarcopenia have a lower tolerance for treatment and treatment-related toxicities. This is consistent with previous reports (26, 29), suggesting that classifying patients according to the presence/absence of sarcopenia may provide a rational basis for identifying patients who are at increased risk of toxicity vs. those who may tolerate treatment well.

Patients with sarcopenia experienced more frequent RT interruptions and subsequently showed inferior treatment outcomes. Many previous studies suggested that interruption or non-completion of RT is significantly associated with patient survival and disease control (30). Known factors affecting RT interruption include depression, anxiety, a lack of appropriate information, pain, and other treatment-related toxicities. Sarcopenia might also prompt patients to discontinue treatment or request a de-intensified or decreased RT dose. Thus, efforts should be made to provide intensive nutritional support, and more education and support are needed to encourage patients not to interrupt treatment (31).

Identifying patients with sarcopenia, especially when accompanied by systemic inflammation, may be valuable for identifying patients at higher risk for poor survival. Many studies have suggested effective strategies to prevent and treat muscle wasting in cancer patients. Specific dietary habits, lifestyle modifications, and treatments that can prevent or delay the negative effects of sarcopenia are recommended. Simple nutritional support can increase muscle mass, and regular exercise can help augment muscle strength (32, 33). Moreover, medications that block the cytokines associated with muscle atrophy signaling pathways (e.g., myostatin/activin, TNFα, and IL-6) or agents that induce muscle hypertrophy signal (e.g., growth hormone agonist, anabolic steroids, and Grelin) may be useful (34). Including these strategies in current anticancer treatments can help extend the survival of cancer patients.

There are several limitations to this study. Because of its retrospective nature, it was not always possible to evaluate all variables and possible confounding factors in all patients. PET-CT or abdominal CT were not retrievable for all subjects, and we could only evaluate routine blood test results; however, other inflammatory markers would likely have provided more informative data. Treatment-related toxicities and tolerances were evaluated retrospectively, causing some ambiguities and missing data. Nevertheless, this study is one of the largest analyses evaluating the prognostic significance of sarcopenia with inflammation in patients with head and neck cancer who received definitive CCRT. Intensive care and nutritional counseling to increase their muscle mass and strength can be provided to the subgroup of patients we identified to be at increased risk of poorer outcomes.

## Conclusion

We found that sarcopenia accompanied by systemic inflammation at initial diagnosis was significantly associated with inferior OS and PFS in patients undergoing definitive CCRT for head and neck cancer. Additionally, patients with sarcopenia required RT interruption more frequently and showed poorer outcomes than those without sarcopenia. To increase treatment tolerance and prevent RT interruption, patients should receive intensive nutritional support and additional types of treatments during CCRT.

## Author contributions

YC, HJ, and IL: study concept and design; KK, CL, and IL: data acquisition and quality control of data. YC, JK, and IL: data analysis and interpretation. YC, IL, and JK: manuscript preparation. HJ, KK, and CL: manuscript review.

### Conflict of interest statement

The authors declare that the research was conducted in the absence of any commercial or financial relationships that could be construed as a potential conflict of interest.
