# Supplementary figures and images for: Prognostic Significance of Sarcopenia With Inflammation in Patients With Head and Neck Cancer Who Underwent Definitive Chemoradiotherapy
Source: Front Oncol. 2018 Oct 22;8:457. doi: 10.3389/fonc.2018.00457 (PMC6232888; doi:10.3389/fonc.2018.00457)

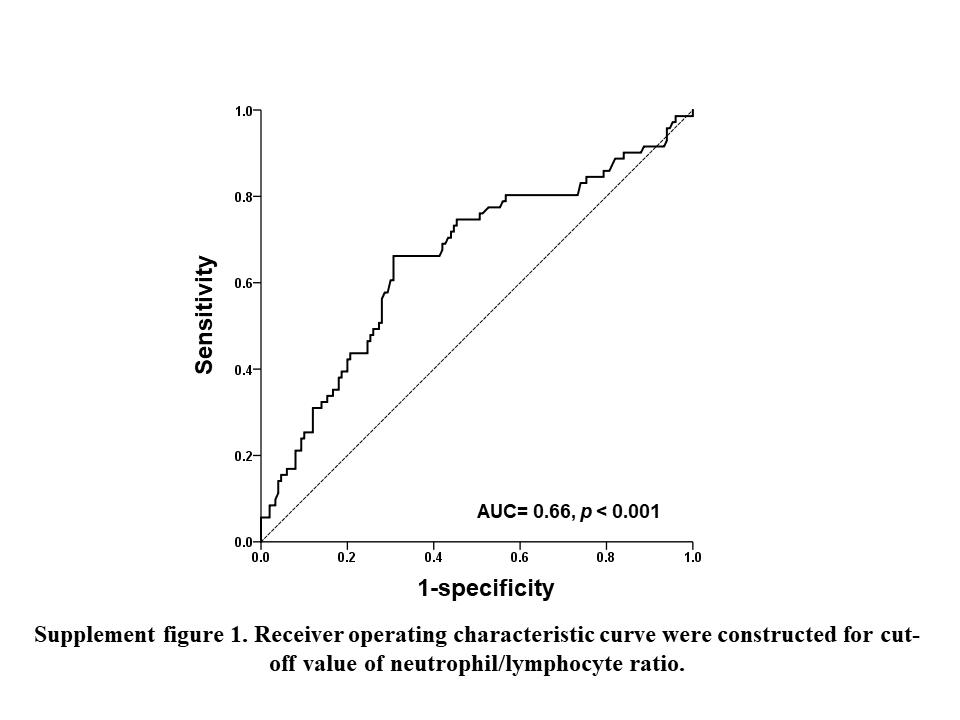

Supplement: Supplementary file 1 [file Image_1.tif]
